# Death Following the Administration of Nitrous Oxide

**Published:** 1897-03

**Authors:** 


### SELECTIONS.

### Death Following the Administration of Nitrous Oxide.

The following case illustrates the influence which nitrous oxide gas may have when administered to persons suffering from atheromatous blood-vessels. A man between fifty and sixty years of age visited the office of a well known dentist who makes a specialty of extracting teeth under the influence of nitrous oxide gas, in order that he might have removed one or two molar teeth which were giving him trouble. He had often taken nitrous oxide gas in the same dentist's office on previous occasions, and always without any ill-effects whatever. On this occasion he took the ordinary quantity, his teeth were extracted, and he returned to consciousness with the usual rapidity. He left the dentist's chair and walked to a washstand, and began to rinse out his mouth with water. While doing this he stated that his right hand felt numb, then complained of the extension of this numbness up his arm, and rapidly to his leg and side. He was helped to a sofa, where, in the course of a very few minutes he became partially unconscious. When I saw him the attack had already been in existence about twenty minutes. He was breathing stertorously, seemed to understand questions put to him, but was unable to answer them clearly, and in the course of a very few minutes passed into absolute insensibility, which, notwithstanding the use of venesection and other measures, deepened into a coma, in which he died about twelve hours after taking the anæsthetic.

This case is reported not as a death due to the direct influence of nitrous oxide gas, but as an instance of the fact that the marked rise in arterial pressure which is produced by the administration of this drug during the period of anæsthesia may cause the rupture of a blood-vessel in persons who have a tendency to apoplexy.—H. A. HARE, M.D., in *Therapeutic Gazette.*

